# Multi-Omics Insights into the Impact of MDH2 on Breast Cancer Progression: A Promising Druggable Target

**DOI:** 10.32604/or.2025.068119

**Published:** 2025-10-22

**Authors:** Botao Pan, Zirun Luo, Xiujuan Yang, Qingqing Liu, Zhimin Yang, Chenglai Xia

**Affiliations:** 1 Scientific Research Center, The Affiliated Foshan Women and Children Hospital, Guangdong Medical University, Foshan, 528000, China; 2 Department of Breast, Thyroid and Head-Neck Surgery, Third Xiangya Hospital of Central South University, Changsha, 410000, China; 3 School of Pharmaceutical Sciences, Southern Medical University, Guangzhou, 515150, China; 4 Jiangsu Natural Active Pharmaceutical Ingredient Design and Separation Technology Engineering Research Center, School of Pharmaceutical Engineering, Jiangsu Food & Pharmaceutical Science College, Huai’an, 223003, China

**Keywords:** Breast cancer, cancer metabolism, malate dehydrogenase 2 (MDH2), druggable target, immunomodulatory metabolites

## Abstract

**Objectives:**

Breast cancer is characterized by significant metabolic dysregulation, in which altered enzyme activity plays a central role. Malate dehydrogenase 2 (MDH2), a key enzyme in the tricarboxylic acid cycle, has been implicated in several malignancies, but its role in breast cancer tumorigenesis and progression remains unclear. We aimed to elucidate the oncogenic role of MDH2 in breast cancer and to evaluate its potential as a diagnostic, therapeutic, and prognostic biomarker.

**Methods:**

We combined *in vitro* cell-based assays with mouse xenograft models to systematically dissect how MDH2 governs breast cancer growth. *In vitro*, we assessed the effects of altered MDH2 expression on proliferation, migration, epithelial–mesenchymal transition (EMT), glucose consumption, and adenosine-5^′^-triphosphate (ATP) production. *In vivo*, we dynamically monitored tumor growth driven by MDH2 overexpression. Transcriptomic profiling, untargeted metabolomics, and *in-silico* druggability analyses were integrated to elucidate downstream mechanisms and therapeutic potential.

**Results:**

*In vitro*, MDH2 depletion suppressed breast cancer cell proliferation and migration, reversed EMT, and markedly reduced glucose consumption and ATP production. *In vivo*, MDH2 overexpression accelerated xenograft tumor growth. Transcriptomic profiling revealed MDH2 had modified the gene expression profile of breast cancer cells, affecting several metastasis-related genes. Kyoto Encyclopedia of Genes and Genomes (KEGG) enrichment analysis identified the phosphatidylinositol 3-kinase (PI3K)/protein kinase B (PKB, also known as AKT) pathway as a downstream effector pathway of MDH2. Untargeted metabolomics uncovered 62 MDH2-regulated metabolites, including the immunomodulatory metabolites adenosine and linoleic acid. *In-silico* modeling confirmed MDH2 as a novel druggable target.

**Conclusion:**

Our findings highlight the role of MDH2 in breast cancer metabolism and suggest it as a promising target for cancer therapies targeting metabolism and tumor growth.

## 1 Introduction

Breast invasive carcinoma (BRCA) remains the most prevalent malignancy in women and a leading cause of cancer-related mortality [1,2]. Despite significant progress in research and clinical outcomes, the incidence and mortality of BRCA are rising sharply in developing countries like China, with worse survival and prognosis compared to developed nations such as the United States [3,4]. This underscores the urgent need for more effective treatments [5,6] and the identification of biomarkers and therapeutic targets.

Breast cancer types include non-invasive in situ and invasive carcinoma, with invasive ductal carcinoma accounting for over 75% of invasive cases [7]. The progression model suggests that invasive breast cancer develops from non-invasive in situ, supported by clinical and molecular evidence [8,9]. During BRCA progression, cancer cells may migrate from ducts or lobules to the bloodstream or lymphatic system, then to distant organs like the liver, lungs, or bones [10,11]. The 5-year survival rate for localized breast cancer has reached 99%, yet it drops sharply to approximately 29% in metastatic breast cancer [12]. Thus, metastatic cell migration is a critical stage in BRCA development. Epithelial-mesenchymal transition (EMT) enhances cell invasion and promotes metastasis, crucial for the benign tumor transformation into invasive tumors [13]. Reprogramming of energy metabolism has been established as a core hallmark of malignancies, including BRCA. Targeting metabolic pathways like glycolysis and the tricarboxylic acid (TCA) cycle is considered a promising approach to reversing EMT in cancer treatment [14]. Therefore, developing metabolic enzymes as potential therapeutic targets to inhibit BRCA invasion and metastasis is essential for understanding the molecular mechanisms of BRCA progression and improving treatment sensitivity.

Malate dehydrogenase (MDH) catalyzes the reversible oxidation of malate to oxaloacetate using the nicotinamide adenine dinucleotide/nicotinamide adenine diphosphate hydride (NAD/NADH) cofactor system [15]. MDH has two key isozymes in eukaryotic cells, MDH1 and MDH2, which are expressed in the cytoplasm and mitochondria, respectively, and plays a vital role in the malate/aspartate shuttle. This shuttle is crucial for transferring cytoplasmic NADH produced during glycolysis into the mitochondria [16,17]. Glycolysis is a vital aspect of cancer cell development, with its rate being essential for adenosine-5^′^-triphosphate (ATP) production and biosynthesis in cancer cells [18,19]. Aberrant MDH2 function has been linked to cancers such as paragangliomas [20], endometrial carcinoma [21], and prostate cancer [22]. MDH2 silencing reduces prostate cancer cell growth and enhances docetaxel sensitivity by causing metabolic inefficiency; high MDH2 expression is associated with shorter recurrence-free survival and treatment tolerance in prostate cancer patients [22]. Several studies have reported the successful development of small-molecule inhibitors targeting MDH2 to inhibit cancer metabolism and tumor growth, which have been applied in colorectal cancer research [23–25]. Modulating tumor metabolism strongly suggests that MDH2 is an innovative and attractive target for cancer treatment. However, the expression pattern, functional role, and molecular mechanism of MDH2 in BRCA have been rarely reported and need further investigation. Identifying MDH2 as a critical regulator of BRCA metastasis could provide additional potential therapeutic drug targets.

With the advent of large-scale technologies, integrating multivariate omics data has become an effective method for identifying therapeutic targets and accelerating cancer drug development. In this study, we integrated data from The Cancer Genome Atlas (TCGA), Genotype-Tissue Expression (GTEx), Human Protein Atlas (HPA), Tumor Immune Estimation Resource (TIMER), transcriptomics, and metabolomics to identify MDH2 as a key metabolic enzyme in BRCA progression. In the present study, we will focus on elucidating the potential role of MDH2 in breast-cancer initiation and progression and on evaluating its feasibility as a novel therapeutic target, thereby providing a theoretical foundation for subsequent mechanistic studies and precision-treatment strategies.

## 2 Materials and Methods

### 2.1 Data Collection, Gene Expression Analysis, and Receiver Operating Characteristic (ROC) Analysis

RNA-seq data, along with associated survival and clinical information, were retrieved from the TCGA database (https://portal.gdc.cancer.gov). Normal human tissue gene mapping data were obtained from the GTEx program (https://commonfund.nih.gov/GTEx). After excluding missing data and duplicate values, the raw data were processed using the transcripts per million (TPM) method and then transformed using log2(TPM+1) for normalization. The analysis was performed using R version 4.2.1. The HPA database (https://www.proteinatlas.org/) provided immunohistochemical (IHC) staining data for MDH2 in breast cancer and normal tissues, as well as RNA expression data for MDH2 in various normal tissues and cancer cell lines. ROC curves for MDH2 were generated using the R packages ‘pROC’ (version 1.18.0) and ‘ggplot2’ (version 3.4.4) to assess its diagnostic value in breast cancer.

### 2.2 Correlation of MDH2 Expression with Survival Prognosis and Clinical Characteristics

Kaplan-Meier plots were used to evaluate the relationship between MDH2 mRNA levels and survival outcomes, including overall survival (OS) and disease-specific survival (DSS). Survival analyses were conducted using the R packages ‘survminer’ (version 0.4.9), ‘survival’ (version 3.4.1), and ‘ggplot2’ (version 3.4.4), with the log-rank test applied (*p* < 0.05). Kaplan-Meier curves for immune infiltration levels and MDH2 gene expression were generated using the Tumor Immune Estimation Resource (TIMER) database (https://cistrome.shinyapps.io/timer/, accessed on 01 August 2025). Additionally, the association between MDH2 mRNA expression and selected clinical phenotypes was examined.

### 2.3 Correlation of MDH2 Expression with Immunity and the Tumor Immune Microenvironment

The single-sample gene set enrichment analysis (ssGSEA) algorithm in the R package gene set variation analysis (GSVA) (v 1.46.0) was employed to analyze the correlation between immune cell infiltration and MDH2 expression levels in TCGA-BRCA using the Spearman correlation test. The algorithm also compared immune infiltration results between high and low (median) MDH2 expression groups using the Wilcoxon rank sum test. Data visualization was performed using the ‘ggplot2’ (version 3.4.4) R package. The ImmuneScore, StromalScore, and ESTIMATEScore were calculated using the ESTIMATE algorithm.

### 2.4 Cells and Antibodies

Human breast cancer cell lines MCF-7 (Cat. No. FH0215), MDA-MB-361 (Cat. No. FH0482), MDA-MB-453 (Cat. No. FH0212), MDA-MB-231 (Cat. No. FH0213), and the normal breast epithelial cell line MCF10A (Cat. No. FH0220) were purchased from Shanghai FuHeng Biotechnology Co., Ltd. (Shanghai, China). All cell lines were authenticated via short tandem repeat (STR) profiling to confirm genetic identity and verified free of mycoplasma contamination. MCF-7 and MCF-10A cells were cultured in Minimal Essential Medium (MEM, Cat. No. FHM01; FuHeng Biology, Shanghai, China) and Mammary Epithelial Cell Medium (MepiCM, Cat. No. 7611; FuHeng Biology, Shanghai, China), respectively, both supplemented with 10% fetal bovine serum (FBS; Gibco, Thermo Fisher Scientific, Grand Island, NY, USA) and 1% penicillin-streptomycin (PS; Merck, Darmstadt, Germany) at 37°C in a humidified 5% CO_2_ atmosphere. MDA-MB-231 cells were cultured in Dulbecco’s Modified Eagle Medium (DMEM; Gibco, Thermo Fisher Scientific, Grand Island, NY, USA) with 10% FBS and 1% PS under the same conditions. MDA-MB-453 cells were cultured in Leibovitz’s L-15 medium (Corning, Corning, NY, USA) with 10% FBS and 1% PS at 37°C without CO_2_ and MDA-MB-361 cells were cultured similarly but with 20% FBS.

Primary antibodies against E-cadherin (Cat. No. 3195), Vimentin (Cat. No. 5741), N-cadherin (Cat. No. 13116), SLUG (Cat. No. 9585), and GAPDH (Cat. No. 5174) were obtained from Cell Signaling Technology (CST; Danvers, MA, USA) and used at 1:1000 dilution. The secondary antibody, HRP-conjugated goat anti-rabbit IgG (Cat. No. 7074) from CST, was applied at 1:3000 dilution. The primary antibody against MDH2 (Cat. No. 15462-1-AP) was from Proteintech Group (Wuhan, China) and used at 1:1000 dilution.

### 2.5 Western Blot Analysis

Protein samples were extracted from human breast cancer cell lines (MCF-7, MDA-MB-361, MDA-MB-453, MDA-MB-231) and the normal breast epithelial cell line MCF10A. Cells were lysed in radioimmunoprecipitation assay (RIPA) buffer (Cat. No. P0038; Beyotime, Shanghai, China) with a protease inhibitor cocktail (Invitrogen, Grand Island, NY, USA) at 0°C, and their concentrations were measured using a bicinchoninic acid (BCA) assay (Cat. No. P0012; Beyotime, Shanghai, China). The samples were separated by sodium dodecyl sulfate-polyacrylamide gel electrophoresis (SDS-PAGE) and transferred to a polyvinylidene fluoride (PVDF) membrane (Millipore, Bedford, MA, USA), which was then blocked in 5% skim milk at room temperature for 2 h. After overnight incubation with primary antibodies at 4°C, the membrane was washed three times with tris-buffered saline with tween 20 (TBST) and incubated with the corresponding secondary antibody for 1 h at room temperature. Protein bands were detected using an enhanced chemiluminescence (Cat. No. 34580; Thermo Fisher Scientific, Waltham, MA, USA) assay and a Champchemiluminometer (Model Smartchemi 610; Sagecreation, Beijing, China). Band intensity was analyzed using ImageJ (version 1.52a) software (National Institutes of Health, USA).

### 2.6 Transfection and Stable Cell Line Establishment

MDH2 siRNA sequences (si-MDH2-1: 5^′^-GCC CAG AAC AAU GCU AAA GUA TT-3^′^; si-MDH2-2: 5^′^-GAA GCC AUG AUC UGC GUC AUU TT-3^′^) were designed and synthesized by Sangon Biotech (Shanghai, China). Transfections were performed using Lipofectamine 3000 (Invitrogen, Grand Island, NY, USA). Stable MDH2-overexpressing (MDH2-OE; MDA-MB-231^MDH2^) and empty-vector control (MDA-MB-231^Vector^) MDA-MB-231 cell lines were both established by Umine Biotechnology Co., Ltd. (Guangzhou, China) using the PiggyBac system. The target gene plasmid and PBase plasmid were co-transfected into cells using the Neon Transfection System (Thermo Fisher Scientific, Waltham, MA, USA). After 48 h, cells were selected with 2 μg/mL puromycin. Total RNA was extracted, and quantitative reverse transcription polymerase chain reaction (qRT-PCR) was performed using MDH2-specific primers.

### 2.7 RNA Extraction and qRT-PCR

Total RNA was extracted from human breast cancer cell lines (MDA-MB-231 and MDA-MB-453) using the FOREGENE reagent (Cat. RE-03014; Chengdu, China) and reverse transcribed into cDNA using PrimeScript RT Master Mix (TaKaRa, Shiga, Japan). qRT-PCR was performed using SYBR green (TaKaRa, Shiga, Japan) with GAPDH as an internal control. The relative expression level was determined using the 2^−ΔΔCt^ method. Primers used were: MDH2 forward, 5^′^-CCGCTGTGAAAGGCTACCTC-3^′^; MDH2 reverse, 5^′^-AATGACGCAGATCATGGCTTC-3^′^; GAPDH (Cat. No. B661104) and beta-actin (Cat. No. B661102) primers were obtained from Sangon Biotech (Shanghai, China).

### 2.8 Cell Viability, Colony Formation, and Live Cell Monitoring Assays

For cell viability assays, 100 μL cell (MDA-MB-231 or MDA-MB-453) suspension (~2000 cells/well) was plated in 96-well plates and incubated for 1, 2, 3 or 4 days post-transfection. Cell viability was measured at 450 nm using a CCK-8 reagent (Cat. No. GK10001; GlpBio, Montclair, CA, USA) and a microplate reader (Model EPOCH2; Biotek, Winooski, VT, USA). For colony formation assays, 700 cells (MDA-MB-231 or MDA-MB-453) were plated in 12-well plates, incubated for two weeks, fixed with 4% paraformaldehyde, stained with 0.2% crystal violet, and counted. Live cell monitoring was performed using the ZenCell Owl Live Cell Dynamic Imaging and Analysis System (Innome, Freiburg, Germany) for four consecutive days post-transfection.

### 2.9 Cell Migration, Apoptosis, and Cell Cycle Assays

For Transwell assays, 15,000 MDA-MB-231 cells in serum-free medium were seeded in the upper chamber of 8 μm Transwell membrane filters (JET BIOFIL, Guangzhou, China), with 600 μL of 10% FBS medium in the lower chamber. After 24 hours, non-migrating cells were removed, and migratory cells were fixed with methanol and stained with 0.1% crystal violet. The migration of cells was imaged using a smartphone (Model SM-S9080; Samsung, Suwon, Republic of Korea).

Apoptosis assay was assessed using the Annexin V/Propidium Iodide (PI) Apoptosis Kit (Cat. No. KGA1102; KeyGEN BioTECH, Nanjing, China) and quantified by flow cytometry. For cell cycle analysis, MDA-MB-231 cells were resuspended in cold PBS (1×, pH 7.2–7.4), fixed in 70% ethanol at 4°C overnight, and then incubated with RNase and PI staining solution for 30 min according to the Cell Cycle Assay Kit protocol (Cat. No. KGA9101; KeyGEN BioTECH, Nanjing, China). The distribution of the cell cycle phases was determined by flow cytometry using FACSCanto II instrument (Becton, Dickinson and Company, Franklin Lakes, NJ, USA), and the data were analyzed with FlowJo software (version 10; Beaverton, OR, USA).

### 2.10 Glucose and ATP Consumption Assays

Glucose and ATP levels in transfected MDA-MB-231 cell samples (2 × 10^6^ cells per sample) were measured using the Glucose Assay Kit (Cat. No. ab65333; Abcam, Cambridge, UK) and the ATP Assay Kit (Cat. No. ab83344; Abcam, Cambridge, UK), respectively, following the manufacturers’ instructions. Absorbance values of the cell samples at 570 nm were measured using a microplate reader, and the results were normalized to the number of cells.

### 2.11 Xenograft Breast Cancer Model

A total of 12 female BALB/c nude mice (4 weeks old, 16~18 g) were purchased from Guangdong Vital River Laboratory Animal Technology Co., Ltd. (Guangzhou, China; Certificate No. SCXK (Yue) 2022-0063). After one week of acclimatization under specific pathogen-free (SPF) environment, the mice were randomly divided into two groups (n = 6 per group) based on body weight. MDA-MB-231 cells (5 × 10^6^) transfected with empty vector or MDH2 overexpression plasmid were suspended in 200 μL PBS (1×, pH 7.2–7.4) and subcutaneously injected into the right flank of mice. Starting from day 10 post-inoculation, tumor dimensions and body weight were recorded every 3 days. All mice were sacrificed on day 31, and tumor tissues were harvested for weighing and photography. Tumor volume was calculated using previously reported formulas [26]. The animal experimental findings were acquired in a blinded manner. The animal study was approved by the Experimental Animal Ethics Committee of Guangdong Medical Laboratory Animal Center (Guangzhou, China), with reference number C202304-5.

### 2.12 Prediction of Potential Binding Sites of MDH2 and Molecular Docking

PyMOL (version 1.8) was utilized to identify putative inhibitor binding sites based on the structure of MDH2 (PDB: 4WLO; https://www.rcsb.org/structure/4WLO). Molecular docking assays were conducted as previously described [27].

### 2.13 Transcriptomics and Untargeted Metabolomics Analysis

Total RNA was extracted from MDA-MB-231 cells (vector control and MDH2-OE groups; 3 pairs of independent replicates) using TRIzol reagent (Cat. No. 15596026CN; Thermo Fisher Scientific, Waltham, MA, USA). Transcriptomic sequencing and data analysis were performed by BioNovoGene Co., Ltd. (Suzhou, China). Briefly, mRNA with a polyA structure was enriched using oligo (dT) magnetic beads and fragmented to approximately 300 bp by ionic disruption. The first strand of cDNA was synthesized using RNA as the template, a 6-base random primer, and reverse transcriptase, followed by synthesis of the second strand of cDNA.

After library construction, PCR amplification was used to enrich the library fragments, which were then selected based on fragment size (450 bp). Libraries were quality controlled using an Agilent 2100 Bioanalyzer, and total and effective library concentrations were tested. Libraries were mixed in proportion to effective library concentration and required data volume, with the libraries containing different index sequences (each sample with a different index, and finally, the downstream data for each sample based on the index). Mixed libraries were diluted to 2 nM and alkali-denatured to form single-stranded libraries. Libraries were sequenced using next-generation sequencing (NGS) on the Illumina HiSeq sequencing platform with paired-end (PE) sequencing. Differentially expressed genes (DEGs) analysis was performed using DESeq, with screening conditions set at |log2FoldChange| > 1 and significance *p*-value < 0.05. Gas chromatography-mass spectrometry (GC-MS) and liquid chromatography-mass spectrometry (LC-MS) analyses were performed by BioNovoGene Co., Ltd., on 1 × 10^7^ vector control and MDH2-OE MDA-MB-231 cells (3 pairs of independent replicates). In our untargeted metabolomics analysis, we calculated the *p* value through statistical tests, determined the Variable Importance in Projection (VIP) value using OPLS-DA, and computed the fold change in metabolite levels. Metabolites with a *p* value less than 0.05 and a VIP value greater than 1 were considered statistically significant and identified as potential biomarkers. The assay protocol and subsequent analysis procedures followed the methods described by Huang et al. [28]. Using R, we leveraged ‘ComplexHeatmap’ (version 2.13.1) and ‘ggplot2’ (version 3.4.4) for multidimensional visualization: the union of differentially expressed genes and samples was subjected to bidirectional clustering and displayed as a heatmap; volcano and pie plots were generated with ggplot2. GO and KEGG enrichment analyses were performed with ‘clusterProfiler’ (version 4.4.4); the top 20 GO terms and pathways with the lowest false discovery rate (FDR), Rich factor, and gene count were selected for presentation.

### 2.14 Statistical Analysis

Statistical differences between two groups were determined using Student’s *t*-test, while one-way ANOVA was used for multiple groups. Correlation matrices were generated using Pearson’s or Spearman’s correlation. All analyses were performed using R software (v 4.2.1) or GraphPad Prism (version 9.0; GraphPad Software LLC, San Diego, CA, USA). Data are presented as mean ± standard deviation (SD), with *p* < 0.05 considered statistically significant; otherwise, marked as not significant (ns).

## 3 Results

### 3.1 MDH2 is Overexpressed in Breast Cancer at Both the mRNA and Protein Levels

**Figure 1:**
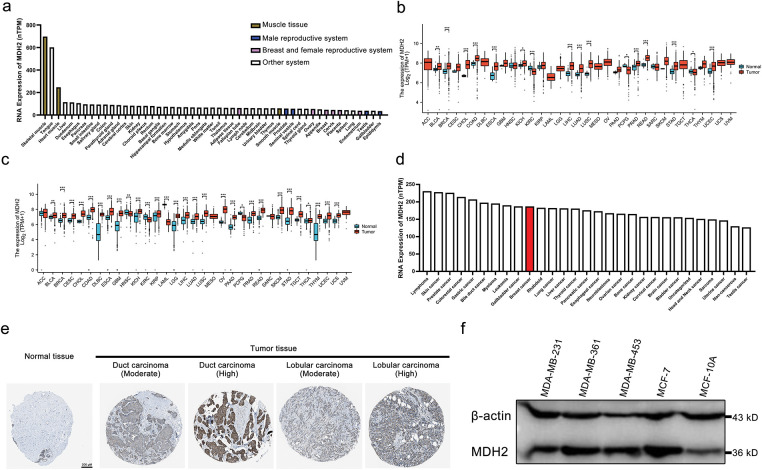
MDH2 expression profile. (**a**) MDH2 mRNA expression in normal tissues, as extracted from the HPA database; (**b**) MDH2 mRNA expression across 33 cancer types in TCGA; (**c**) Differential MDH2 mRNA expression between TCGA cancers and normal tissues from the GTEx; (**d**) MDH2 mRNA expression in various tumor cell lines, as determined by the HPA database; (**e**) Representative IHC images of MDH2 in normal and malignant breast tissues; (**f**) MDH2 protein expression levels in breast cancer cell lines, as assessed by Western blot assay. *p < 0.05, **p < 0.01, and ***p < 0.001

To investigate the expression profile of MDH2 in healthy tissues, we analyzed data from the HPA database. MDH2 is highly expressed in human muscle tissues, including heart muscle, skeletal muscle, and smooth muscle, but is lowly expressed in the male reproductive system (prostate, seminal vesicle, testis, and epididymis) as well as in the breast and female reproductive system (fallopian tube, ovary, breast, cervix, placenta, and endometrium) (Fig. 1a). We next evaluated the expression pattern of MDH2 mRNA across various cancers using data from TCGA. MDH2 mRNA was significantly upregulated in 13 types of cancer (cholangiocarcinoma (CHOL), colon adenocarcinoma (COAD), bladder urothelial carcinoma (BLCA), breast invasive carcinoma (BRCA), esophageal carcinoma (ESCA), kidney chromophobe (KICH), liver hepatocellular carcinoma (LIHC), lung adenocarcinoma (LUAD), lung squamous cell carcinoma (LUSC), prostate adenocarcinoma (PRAD), rectum adenocarcinoma (READ), stomach adenocarcinoma (STAD), and uterine corpus endometrial carcinoma (UCEC)) and significantly downregulated in 3 types of cancer (kidney renal clear cell carcinoma (KIRC), pheochromocytoma and paraganglioma (PCPG), and thyroid carcinoma (THCA)) (Fig. 1b). To further analyze MDH2 expression patterns, we combined data from the TCGA and GTEx databases, which revealed that MDH2 mRNA was significantly highly expressed in 25 cancer types, including BRCA, and significantly less expressed in 4 cancer types (Fig. 1c). Using the HPA database, we also assessed MDH2 mRNA levels in 29 distinct tumor cell lines and found that MDH2 was highly expressed in all of these cell lines, with particularly high levels in breast cancer (Fig. 1d).

At the protein level, immunohistochemical analysis from the HPA database showed that MDH2 protein was markedly overexpressed in breast cancer tissues (ductal and lobular carcinomas) compared to normal breast tissue (Fig. 1e). Consistent with these findings, Western blot analysis revealed that MDH2 protein expression was low in normal breast epithelial cells (MCF10A) but elevated in various breast cancer cell lines (Fig. 1f). Collectively, these results indicate that MDH2 is overexpressed in breast cancer at both the transcriptional and translational levels, suggesting that the MDH2 gene plays a crucial role in breast cancer pathogenesis.

### 3.2 Correlation of MDH2 Expression with Clinicopathological Characteristics and Prognostic Value in BRCA

**Figure 2:**
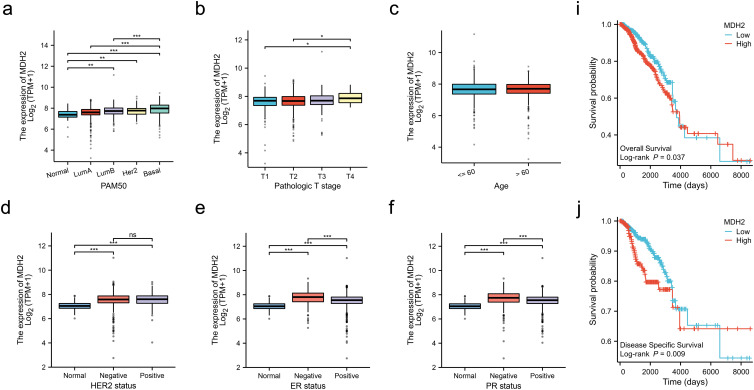

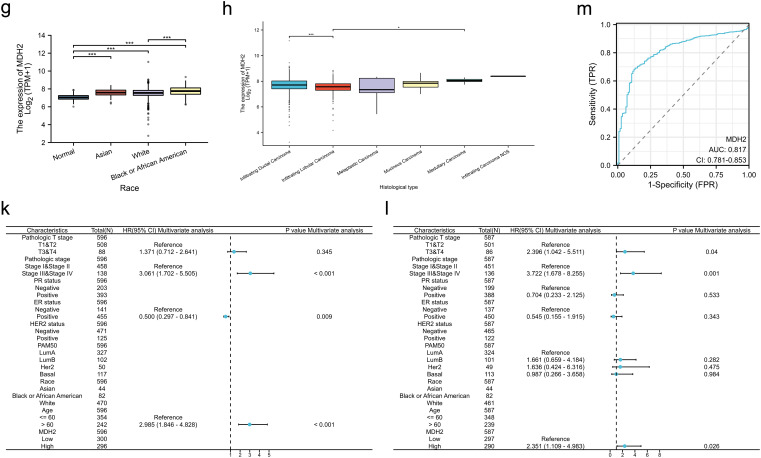
Correlation of MDH2 expression with clinicopathological characteristics and prognostic value in BRCA. (**a–h**) Correlation analysis of MDH2 mRNA levels with PAM50 subtype (**a**), pathological T stage (**b**), age (**c**), HER2 status (**d**), ER status (**e**), PR status (**f**), race (**g**), and histological type (**h**) in TCGA-BRCA patients; (**i,j**) Kaplan-Meier analysis of the association between MDH2 mRNA expression and OS (**i**) or DSS (**j**); (**k,l**) Multivariate Cox analyses of MDH2 and pathological characteristics in predicting OS (**k**) and DSS (**l**). (**m**) ROC curve analysis of the prognostic model. *p < 0.05, **p < 0.01, and ***p < 0.001

Analysis of BRCA samples from various clinical subtypes in the TCGA revealed substantial correlations between MDH2 transcript levels and breast cancer subtype, pathological T stage, human epidermal growth factor receptor 2 (HER2) status, estrogen receptor (ER) status, progesterone receptor (PR) status, race, and histological type. As shown in Fig. 2a, MDH2 expression was highest in the basal-like breast cancer subtype, followed by the Luminal-B and Luminal-A subtypes. Data from the TCGA database indicate that MDH2 expression is abnormally elevated in advanced breast cancer (Fig. 2b). Additionally, no correlation was found between MDH2 and patient age (Fig. 2c). MDH2 expression levels in breast malignancies with varying receptor status have implications for treating breast cancer. As demonstrated in Fig. 2d–f, MDH2 mRNA levels were higher in the negative type, particularly in the estrogen receptor (ER) (-) and progesterone receptor (PR) (-), regardless of the receptor status. Moreover, black and African American BRCA patients had higher MDH2 expression levels than white BRCA patients (Fig. 2g). Among breast cancers of different histological types, infiltrating ductal carcinoma had higher MDH2 expression than infiltrating lobular carcinoma (Fig. 2h).

**Figure A1:**
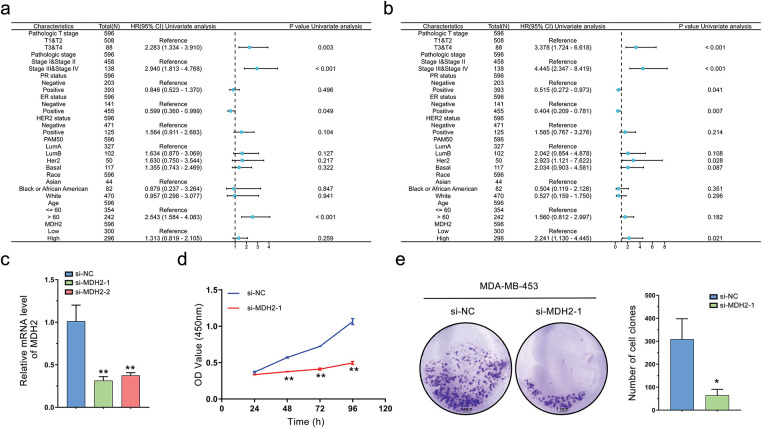
Univariate Cox analysis of MDH2 and its impact on the proliferation of MDA-MB-453 Cells. (**a**,**b**) Univariate Cox analysis of MDH2 and pathological characteristics in predicting OS (**a**) and DSS (**b**); (**c**) Efficacy of siRNA knockdown was evaluated by qRT-PCR (n = 3); (**d**) Impact of MDH2 knockdown on cell viability in MDA-MB-453 cells assessed by CCK-8 assay (n = 3); (**e**) Effect of MDH2 knockdown on cell proliferation evaluated by colony formation assays (n = 3). Data are presented as mean ± SD. *p < 0.05, **p < 0.01

To elucidate the prognostic significance of MDH2 in breast cancer, we stratified patients into high and low MDH2 expression groups and assessed their OS and DSS. Kaplan-Meier survival analysis revealed that elevated MDH2 expression was significantly associated with inferior OS and DSS (Fig. 2i,j). Further, univariate and multivariate Cox regression analyses confirmed MDH2 as an independent prognostic factor for DSS (Figs. 2k,l and A1a,b). Additionally, ROC analysis, which measures the accuracy of a marker to discriminate between outcomes, showed an area under the curve (AUC) of 0.817, highlighting MDH2’s robust predictive capacity for prognosis (Fig. 2m). Collectively, these findings implicate MDH2 as a likely oncogene in BRCA.

### 3.3 MDH2 Expression Is Associated with Immune Infiltration Levels

**Figure 3:**
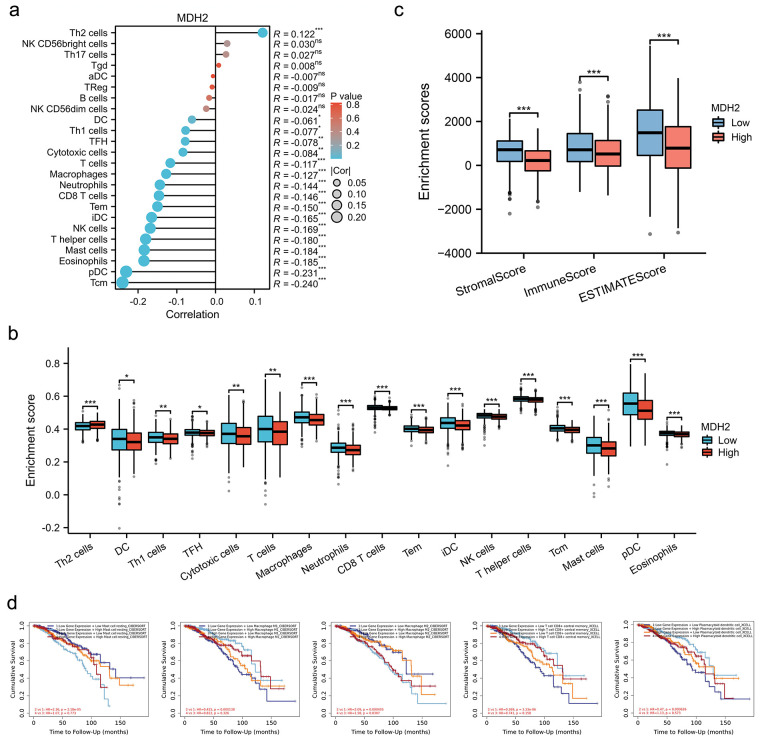
Correlation between MDH2 expression and immune cell infiltration. (**a**) Correlation between MDH2 expression and various immune cell infiltrations depicted in a lollipop plot; (**b**) Correlation between MDH2 high or low expression subgroups and scores of 17 immune cell infiltrations; (**c**) ImmuneScore, StromalScore, and ESTIMATEScore correlate with MDH2 expression in BRCA; (**d**) Survival analysis of combinations of MDH2 expression and different immune cell scores in BRCA. Not significant (ns); *p < 0.05, **p < 0.01, and ***p < 0.001

While existing evidence underscores the prognostic significance of MDH2 in BRCA, its potential role in the context of immune infiltration merits further exploration. As a crucial component of the tumor microenvironment, the number of immune cells infiltrating the tumor is correlated with cancer initiation, progression, and metastasis [29,30]. MDH2 expression significantly correlated positively with Th2 cells and negatively with DC, Th1 cells, TFH, cytotoxic cells, T cells, macrophages, neutrophils, CD8 T cells, Tem, iDC, NK cells, T helper cells, mast cells, eosinophils, pDC, and Tcm (Fig. 3a). BRCA patients were then divided into two subgroups with high and low MDH2 expression to assess immune infiltration enrichment. The high MDH2 expression subgroup exhibited reduced enrichment scores in most immune cells, with the exception of Th2 cells (Fig. 3b). To further investigate the role of MDH2 in tumor immune infiltration, the StromalScore, ImmuneScore, and ESTIMATEScore were integrated across BRCA with MDH2 expression. The results revealed a negative correlation between MDH2 expression and these three categories of ESTIMATES (Fig. 3c).

The Cox proportional hazards model was then employed to further explore the clinical significance of MDH2 expression in tumor immune subgroups. The TIMER database showed that different levels of immune cell infiltration and MDH2 expression in BRCA were associated with distinct survival outcomes. In the group with low MDH2 expression, high levels of T cell CD8(+), macrophage M1, macrophage M2, and mast cell resting infiltration were associated with decreased BRCA survival (Fig. 3d). Conversely, in the group with elevated MDH2 expression, high levels of macrophage M2 infiltration were associated with poor BRCA survival. These findings suggested that survival in BRCA patients with varying levels of MDH2 expression is substantially related to the number of immune cells that infiltrate the tumor.

### 3.4 Knockdown of MDH2 Inhibits Proliferation of Breast Cancer Cells In Vitro

**Figure 4:**
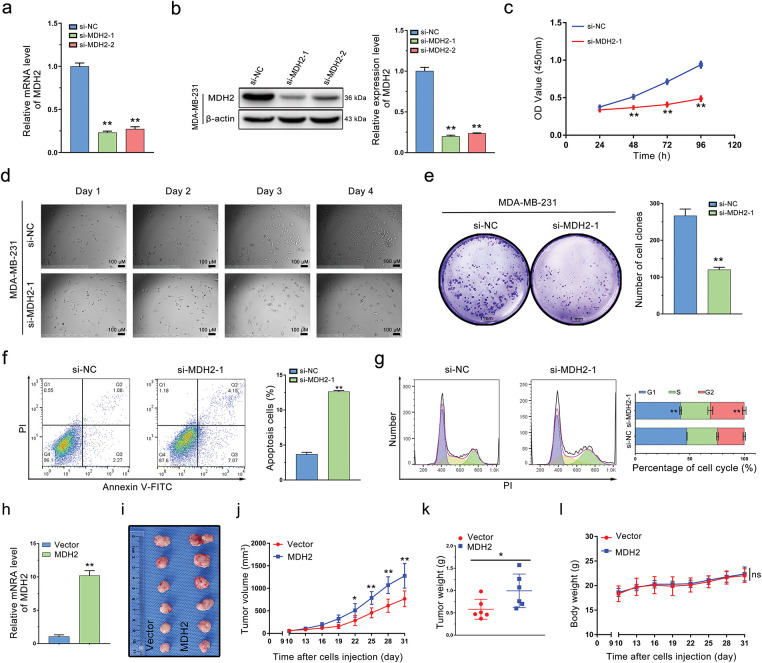
Oncogenic role of MDH2 in breast cancer. (**a**,**b**) Efficacy of siRNA knockdown was evaluated by qRT-PCR (**a**) and Western blotting (**b**) (n = 3); (**c**) Impact of MDH2 knockdown on cell viability in MDA-MB-231 cells assessed by CCK-8 assay (n = 3); (**d**) Influence of MDH2 knockdown on morphology and proliferation of MDA-MB-231 cells determined by real-time monitoring; (**e**) Effect of MDH2 knockdown on cell proliferation evaluated by colony formation assays (n = 3); (**f,g**) Consequences of MDH2 knockdown on cell apoptosis (**f**) and cell cycle (**g**) in MDA-MB-231 cells measured by flow cytometry (n = 3); (**h**) qRT-PCR analysis of MDH2 overexpression efficiency in MDA-MB-231 cells; (**i**) Representative images of xenograft tumors excised from nude mice (n = 6); (**j**) Tumor growth curves in nude mice injected with MDA-MB-231 cells (n = 6); (**k**) Weight of xenograft tumors from mice injected with MDA-MB-231 cells (n = 6); (**l**) Body weight change curve of mice (n = 6). Data are presented as mean ± SD. *p < 0.05, **p < 0.01, and ^ns^*p* > 0.05

To explore the role of MDH2 in breast cancer, we used two distinct small interfering RNAs (si-MDH2-1 and si-MDH2-2) to transiently suppress MDH2 expression in MDA-MB-231 and MDA-MB-453 cells. After confirming the silencing efficacy of siRNA by qRT-PCR (Figs. 4a and A1c) and Western blotting (Fig. 4b), we assessed the effects of MDH2 knockdown on cell proliferation, apoptosis, and cell cycle progression.

As shown in Fig. 4c, MDA-MB-231 cells with reduced MDH2 expression exhibited inhibited proliferation compared to the si-NC group. We monitored cell growth over four consecutive days and observed that cells with transient MDH2 depletion grew more slowly than control cells and displayed morphological changes (Fig. 4d). Additionally, we evaluated the impact of MDH2 on cell proliferation using a clone formation assay. The number of cell clones in the si-MDH2 group was significantly lower than in the control group (Fig. 4e). Notably, analogous inhibitory effects were corroborated in MDA-MB-453 cells via CCK-8 assays and clonogenic experiments (Fig. A1d,e).

We further examined the effects of MDH2 inhibition on apoptosis and the cell cycle using flow cytometry. As depicted in Fig. 4f, MDH2 knockdown in MDA-MB-231 cells significantly increased the proportion of apoptotic cells. Moreover, MDH2 reduction altered the cell cycle distribution and induced sub-G2 phase arrest (Fig. 4g). Collectively, these results indicate that MDH2 suppression inhibits the proliferation of breast cancer cells.

### 3.5 MDH2 Overexpression Promotes Breast Cancer Tumor Growth In Vivo

To investigate the impact of MDH2 on breast cancer tumor proliferation *in vivo*, we engineered MDH2-OE MDA-MB-231 cells. As predicted, these cells exhibited a significant increase in MDH2 mRNA expression (Fig. 4h). To evaluate the role of MDH2 in breast cancer tumorigenesis *in vivo*, we subcutaneously implanted MDH2-OE or vector-infected MDA-MB-231 cells into nude mice. After 31 days, tumors derived from MDH2-OE cells were noticeably larger than those from vector control cells (Fig. 4i). Additionally, both the size and weight of xenograft tumors originating from MDH2-OE cells were significantly greater than those from control cells (Fig. 4j,k). Throughout the experiment period, no fatalities or significant changes in body weight were observed in either group (Fig. 4l). Collectively, these findings suggest that MDH2 overexpression enhances the growth and proliferation of breast cancer tumors.

### 3.6 Transcriptomics Profiling of MDH2 Overexpression in MDA-MB-231 Cells

**Figure 5:**
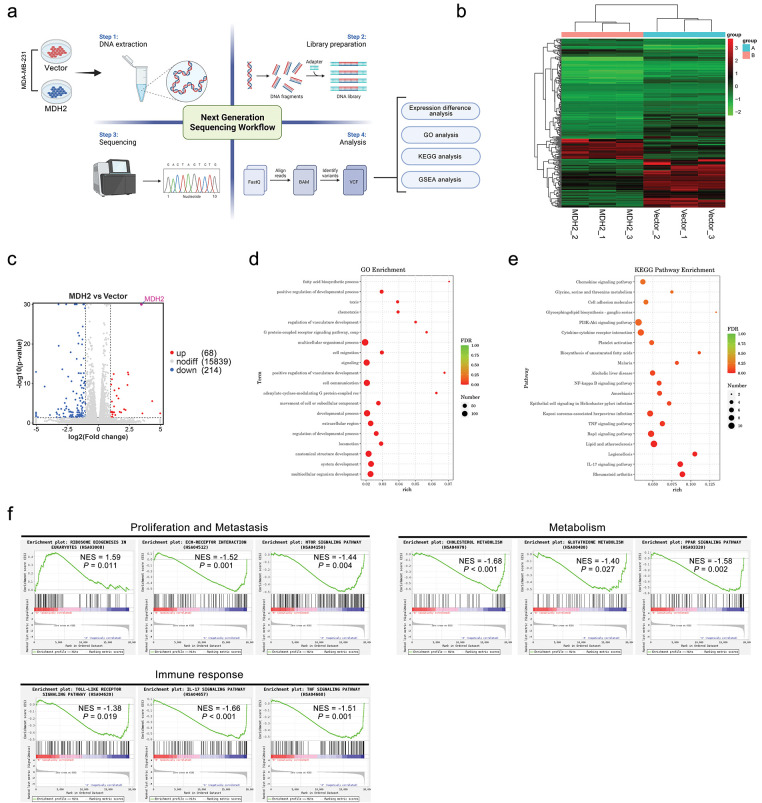
RNA-seq analysis of downstream genes and pathways regulated by MDH2. (**a**) Schematic overview of RNA-seq analyses conducted in this study; (**b**) Hierarchical clustering of differentially expressed genes identified through RNA-seq analysis (n = 3); (**c**) Volcano plot depicting differentially regulated gene expression between control and MDH2-OE cells from RNA-seq analysis; (**d,e**) GO (**d**) and KEGG (**e**) pathway analysis of differentially expressed genes in the MDH2-OE transcriptome; (**f**) Enrichment plots from GSEA. GSEA highlights differentially expressed genes in response to MDH2 associated with ribosome biogenesis in eukaryotes, ECM-receptor interaction, mTOR signaling pathway, cholesterol metabolism, glutathione metabolism, PPAR signaling pathway, toll-like receptor signaling pathway, IL-17 signaling pathway, and TNF signaling pathway

To further elucidate the downstream molecular mechanisms underlying MDH2’s effects on tumor growth, we conducted RNA sequence analysis to investigate the transcriptome of MDH2-OE MDA-MB-231 cells (Fig. 5a). Compared with the vector control group, a total of 282 differentially expressed genes were identified, including 68 upregulated and 214 downregulated genes (|log2FoldChange| > 1, *p* < 0.05) (Supplementary file 1). Cluster analysis of DEGs was performed using a heatmap (Fig. 5b), and the fold changes of DEGs were visualized using Volcano plots (Fig. 5c). Notably, MDH2 was among the most significantly upregulated genes (approximately 11.21-fold change), consistent with our qRT-PCR results and confirming the successful establishment of the MDH2-OE cell line. Interestingly, MDH2 overexpression upregulated several genes associated with breast cancer metastasis (IL13RA2, MMP3, PTGS1, SYK, VCAN, and FLT1) and downregulated several genes associated with metastasis suppression (NOTCH3 and HTRA3).

To gain insight into the biological processes underlying MDH2-driven tumor progression, we identified significantly overrepresented gene ontology (GO) terms for clusters of DEGs. As shown in Fig. 5d, overexpression of MDH2 in MDA-MB-231 cells enhanced responses related to the positive regulation of developmental process, cell migration, positive regulation of vasculature development, movement of cell or subcellular component, and locomotion. To further explore the relationship between DEGs and pathways, we conducted the pathway-based analysis using the Kyoto Encyclopedia of Genes and Genomes (KEGG) database. KEGG enrichment analysis revealed that the majority of DEGs (LPAR5, SYK, NOS3, FLT1, COL6A2, VEGFD, THBS2, GNG2, SGK2, DDIT4, and IL3RA) were enriched in the “PI3K-Akt signaling pathway” (Fig. 5e). Additionally, MDH2 overexpression was associated with several signaling pathways linked to cell proliferation and migration, including “cell adhesion molecules”, “NF-kappa B signaling pathway”, “TNF signaling pathway”, “Rap1 signaling pathway”, and “IL-17 signaling pathway”.

To further investigate which signaling pathways were regulated by MDH2 overexpression, we performed gene set enrichment analysis (GSEA) on all altered genes. GSEA revealed that MDH2-related genes were primarily enriched in pathways related to metabolic, cell proliferation and metastasis, and immune response (Fig. 5f). In eukaryotes, high MDH2 expression is mainly associated with ribosome biogenesis, while low MDH2 expression is associated with pathways such as the toll-like receptor signaling pathway, IL-17 signaling pathway, TNF signaling pathway, ECM-receptor interaction, mTOR signaling pathway, cholesterol metabolism, glutathione metabolism, and PPAR signaling pathway. These findings suggest that MDH2 may regulate tumor proliferation through multiple mechanisms.

### 3.7 Knockdown of MDH2 Inhibits Breast Cancer Cell Migration and Disrupts Glycolysis

**Figure 6:**
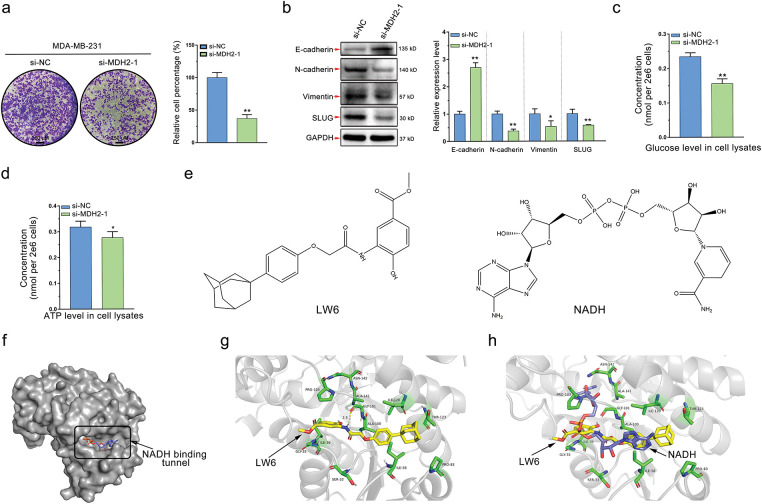
MDH2 as a druggable target that prevents cancer cell migration and disrupts glycolysis. (**a**) Impact of MDH2 knockdown on the migration of MDA-MB-231 cells assessed by Transwell assay; (**b**) Western blot analysis of EMT markers following MDH2 knockdown in MDA-MB-231 cells. Relative expression levels of E-cadherin, N-cadherin, vimentin, and slug were normalized to beta-actin; (**c,d**) Effects of MDH2 knockdown on glucose (**c**) and ATP (**d**) levels in MDA-MB-231 cells, measured using respective assay kits; (**e**) Chemical structures of LW6 (a small molecule MDH inhibitor) and NADH (the natural ligand for MDH2); (**f**) Crystal structure of MDH2 protein (PDB: 4WLO) visualized using PyMOL, highlighting the tunnel where NADH binds as a potential drug-binding site; (**g**) Optimal binding mode of LW6 (yellow) to MDH2 (gray) simulated by molecular docking (−9.8 kcal/mol). Amino acid residues (green) surrounding the binding tunnel are labeled, with yellow dashed lines indicating hydrogen bonds; (**h**) Superimposition of LW6 (yellow) with NADH (purple) in the original co-crystal structure, with surrounding amino acid residues (green) labeled. Data are expressed as mean ± SD. *p < 0.05, and **p < 0.01

Given the transcriptomic data suggesting MDH2’s involvement in cell migration and metabolism, we explored the migratory and metabolic functions of MDH2 in breast cancer. Using Transwell assays, we evaluated the impact of MDH2 suppression on cell motility. As shown in Fig. 6a, MDH2 knockdown significantly inhibited the migration of MDA-MB-231 cells. Given that the EMT is a key event in tumor migration initiation, we examined the effect of MDH2 on EMT markers in MDA-MB-231 cells. Western blot analysis confirmed that suppression in MDA-MB-231 cells significantly upregulated E-cadherin expression while downregulating slug, N-cadherin, and vimentin expression (Fig. 6b). These findings suggest that MDH2 reduction inhibits malignant progression of MDA-MB-231 breast cancer cells.

MDH2, a key enzyme in the TCA, likely impacts glycolysis in breast cancer cells. Elevated glycolysis is a prevalent and dominant characteristic of cancer metabolism, with dysregulation of metabolic enzymes playing a significant role. To further validate this hypothesis, we conducted glucose and ATP consumption assays, well-known indicators of the Warburg effect. As anticipated, MDH2 knockdown significantly decreased glucose uptake and ATP production in MDA-MB-231 cells, indicating that MDH2’s biological function is essential for glycolysis (Fig. 6c,d).

### 3.8 In Silico Identification of MDH2 as a Druggable Target

Our experimental findings suggest that MDH2 is a potential drug target for breast cancer treatment. Naik et al. [31] identified MDH2, a mitochondrial enzyme, as a direct target of the HIFα inhibitor LW6. Given this, MDH2 inhibitors could be designed based on the structure of LW6. In this study, we used LW6 as a small molecule regulator of MDH2 (Fig. 6e) to further validate MDH2 as a druggable target. To identify the most likely binding site for the inhibitor, we analyzed the crystal structure of MDH2 *in silico*. We hypothesized that the NADH-binding tunnel is the most plausible binding site (Fig. 6f). Molecular docking revealed that the binding energy of LW6 to the NADH binding tunnel of MDH2 was −9.8 kcal/mol (Fig. 6g). When superimposed on the NADH molecule in the original PDB entry (4WLO), LW6 partially overlapped with NADH, indicating that LW6 binds to MDH2 in a manner comparable to NADH (Fig. 6h). Consistent with previous findings [25], LW6 likely acts as an NADH-competitive ligand for MDH2, interfering with MDH2^′^s biological function in an NADH-competitive inhibitory mode. This supports the notion that MDH2 is a promising druggable target.

### 3.9 Metabolomic Profiling of MDH2 Overexpression in MDA-MB-231 Cells

**Figure 7:**
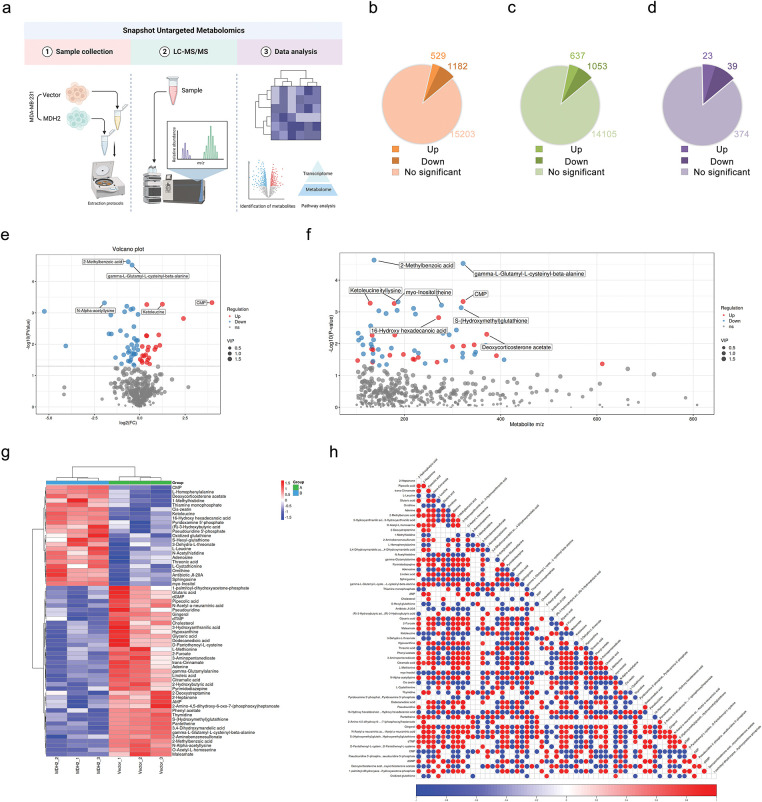
Metabolic profiling in MDH2-OE cells. (**a**) Schematic of the experimental design for untargeted global metabolomics in MDH2-OE and control MDA-MB-231 cells; (**b,c**) A pie chart shows the number of differential metabolites identified from the MS primary metabolite profiles in positive (**b**) or negative (**c**) ion mode (n = 3) (*p* < 0.05, VIP > 1); (**d**) A pie chart shows the number of differential metabolites identified from the MS secondary metabolite profile (n = 3) (*p* < 0.05, VIP > 1); (**e**) Volcano plot of differential metabolites between MDH2-OE and control cells; (**f**) Scatter plot of differential metabolite ratios vs. *p*-values. Each point on the plot has a horizontal coordinate representing the mass-to-charge ratio of the corresponding metabolite and a vertical coordinate representing the logarithmic value of –log10 of the *p* value; (**g**) Hierarchical clustering heat map of 62 differential metabolites; (**h**) Heat map of differential metabolite associations. The vertical and diagonal coordinates reflect the names of the differential metabolites, and the colors represent the correlations, with red being positively correlated and blue being negatively linked. The darker the color, the higher the correlation

To explore how MDH2 expression modifies the metabolic profiles of breast cancer cells, we extracted polar metabolites from vector control and MDH2-OE MDA-MB-231 cell samples and performed untargeted LC-MS metabolomics analysis, as outlined in the flowchart (Fig. 7a). In the primary metabolite profiles, we identified a total of 1711 significant differential metabolites (529 upregulated and 1182 downregulated) in positive ion mode (Fig. 7b, Supplementary file 2) and 1690 significant differential metabolites (637 upregulated and 1053 downregulated) in negative ion mode (Fig. 7c, Supplementary file 3), using the screening criteria *p* < 0.05 and VIP > 1. The secondary metabolite profile consisted of 23 significantly upregulated and 39 significantly downregulated metabolites (Supplementary file 4), as shown in Fig. 7d. A volcano plot illustrates the distribution of differential metabolites between the two groups, with the top five metabolites with the smallest *p* value highlighted (2-Methylbenzoic acid, gamma-L-Glutamyl-L-cysteinyl-beta-alanine, CMP, N-Alpha-acetyllysine, and Ketoleucine) (Fig. 7e). Scatter diagrams based on the mass-to-charge ratio and *p* value of metabolites were generated to better understand the distribution of metabolites in the samples (Fig. 7f). Additionally, hierarchical clustering analysis was used to determine the metabolic patterns of significantly distinct metabolites between groups, displayed as heatmap (Fig. 7g). We then performed differential metabolite association analysis to investigate trends among metabolites, calculating the Pearson correlation coefficient between all metabolites to analyze the correlation between each metabolite (Fig. 7h). These results indicated that alterations in MDH2 expression significantly altered the metabolic profiles of breast cancer cells compared to the vector controls, suggesting a complex metabolic mechanism.

**Figure 8:**
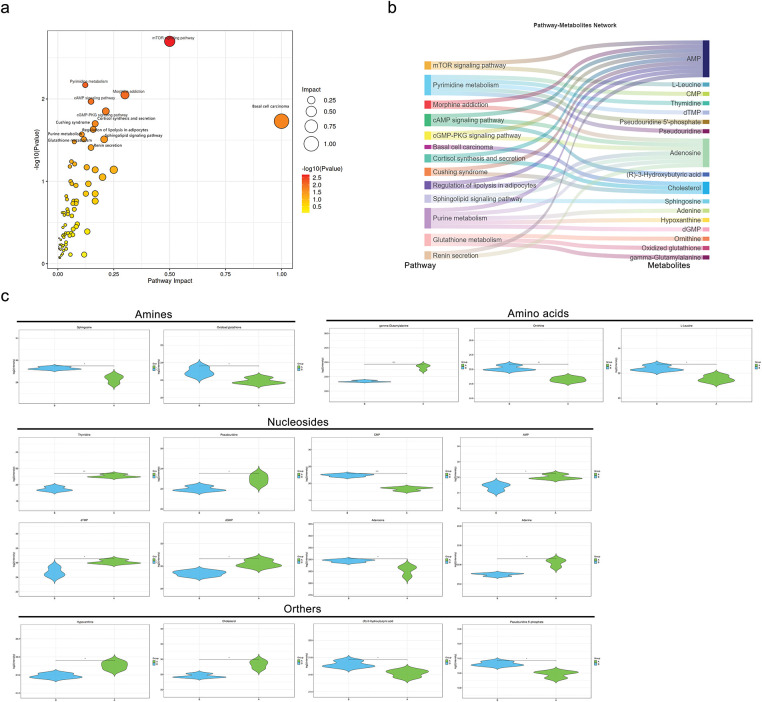
Metabolic pathway analysis and pathway-metabolite network based on differential metabolites. (**a**) Bubble diagram of metabolic pathways and their impact factors. Each point represents a metabolic pathway, with the horizontal axis indicating the impact value (enrichment in various metabolic pathways) and the vertical axis representing the –log10 (*p*) value, where the *p* derived from the hypergeometric distribution test; (**b**) Network diagram linking pathways and metabolites. Differential metabolites are mapped to their enriched pathways (left) and the metabolites involved in these pathways are shown on the right; (**c**) Violin plots of depicting the intensity levels of representative metabolites, including amines, amino acids, nucleosides, and others (n = 3). *p < 0.05, **p < 0.01, and ***p < 0.001

Further investigation into the metabolic pathways involving these 62 significantly different metabolites revealed ten altered metabolic pathways (Fig. 8a), with the top five being the mTOR signaling pathway, pyrimidine metabolism, morphine addiction, cAMP signaling pathway, and cGMP-PKG signaling pathway. A network of pathways and metabolites was constructed to determine which metabolites were implicated in the top 10 pathways. As illustrated in Fig. 8b, 17 metabolites were involved in modifying these pathways, with AMP, adenosine, and cholesterol being the most involved. Quantitative analysis of these metabolites in MDA-MB-231 cells between the control (A) and MDH2 overexpression (B) groups is shown in Fig. 8c. Most of these altered metabolites were classified as adenosine, followed by amino acids and amines.

### 3.10 Integrated Transcriptomics and Metabolomics Analysis

**Figure 9:**
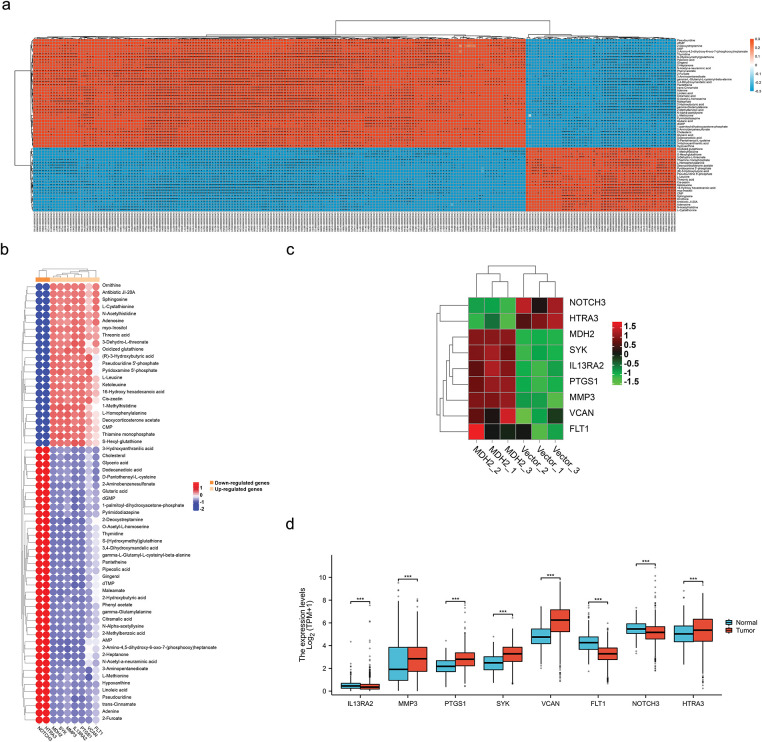
Integrated transcriptomics and metabolomics analysis. (**a**) Correlation between transcriptomics and metabolomics data was assessed using Pearson’s Correlation. Each square represents the correlation and significance between a transcript and a metabolite, with the magnitude of the correlation coefficients indicated by different colors and shades. Red denotes positive correlation, blue denotes negative correlation, and darker colors represent higher correlation coefficients; (**b**) Pearson Correlation analysis was performed to determine the correlation between selected DEGs (Genes related to breast cancer metastasis) and 62 metabolites; (**c**) Hierarchical clustering heatmap of breast cancer metastasis-related DEGs derived from RNA-seq analysis results; (**d**) Differential expression of breast cancer metastasis-related DEGs mRNA in TCGA cancer and GTEx normal tissues. Significance is marked with an asterisk: *p < 0.05, **p < 0.01, and ***p < 0.001

To further elucidate how MDH2 promotes breast cancer progression from a metabolic standpoint, we integrated metabolomic and transcriptomic data to analyze the changes in relevant molecular profiles of MDH2 when regulating progression. Based on the results of metabolomic and transcriptomic analyses, which identified differentially expressed metabolites and transcripts, we performed Pearson^′^s correlation analysis to determine the correlations between these two groups and their *p* values, and generated a heatmap. As shown in Fig. 9a, significant positive or negative correlations were observed between metabolites and up- and down-regulated genes.

Given that MDH2 is implicated in cell migration and metabolism, we conducted correlation analyses on DEGs associated with breast cancer metastasis and 62 differential metabolites to systematically and comprehensively disclose the regulatory mechanisms of MDH2 on these biological processes. Consistent with the correlation pattern between MDH2 and metabolites, the results demonstrated that DEGs (FLT1, VCAN, PTGS1, IL13RA2, MMP3, and SYK) associated with promoting breast cancer metastasis were positively correlated with 23 different metabolites and negatively correlated with 39 different metabolites (Fig. 9b). In contrast, DEGs associated with inhibiting breast cancer metastasis (HTRA3 and NOTCH3) were negatively correlated with 23 metabolites and positively correlated with 39 metabolites. Additionally, Fig. 9c presents a heatmap of the transcriptomic expression of DEGs linked to breast cancer metastasis. Based on the TCGA and GTEx databases, we evaluated the expression patterns of these DEGs in breast cancer. HTRA3, SYK, MMP3, PTGS1, and VCAN mRNA expression were significantly upregulated in BRCA relative to normal tissue (Fig. 9d), whereas NOTCH3, IL13RA2, and FLT1 mRNA expression were significantly downregulated.

## 4 Discussion

Recent studies have unveiled the critical role of MDH2 in various cancers, including glioblastoma [32], endometrial carcinoma [33], and ovarian cancer [34]. Notably, high MDH2 expression has been associated with shorter recurrence-free survival and increased chemotherapy tolerance in prostate cancer patients. Mechanistically, MDH2 silencing has been shown to inhibit prostate cancer cell proliferation and enhance docetaxel sensitivity by inducing metabolic inefficiency [22]. These findings underscore the potential of MDH2 as an innovative and desirable target for cancer therapy. However, the function of MDH2 in breast cancer remains largely unexplored, necessitating further investigation.

In this study, we leveraged gene expression profiling and comprehensive analysis of TCGA data to elucidate the role of MDH2 in breast cancer. Our results revealed that MDH2 is abnormally overexpressed at both the mRNA and protein levels in breast cancer tissue samples. Intriguingly, MDH2 transcript levels in breast cancer patients were strongly correlated with various clinicopathological features, including breast cancer subtype, pathological T stage, HER2 status, ER status, PR status, race, and histological type. Specifically, MDH2 expression was significantly elevated in patients with advanced breast cancer compared to those with early-stage disease, and it was also higher in patients with ER (-) and PR (-) status. Kaplan-Meier survival analysis further demonstrated that elevated MDH2 expression is strongly associated with poor prognosis in breast cancer patients. Collectively, these findings led us to hypothesize that MDH2 may exert a pro-oncogenic function in breast cancer and could serve as a biomarker for diagnosis, treatment, and prognosis in breast cancer patients.

To validate the oncogenic role of MDH2 in breast cancer, we conducted a series of *in vitro* and *in vivo* experiments. Our *in vitro* studies demonstrated that suppressing MDH2 expression in breast cancer cells significantly induced apoptosis, inhibited cell proliferation, altered cell cycle distribution, and induced G2 phase arrest. Consistent with these findings, *in vivo* experiments revealed that transplantation of MDA-MB-231 cells overexpressing MDH2 into nude mice resulted in tumors with significantly larger volumes compared to controls. These results are in line with previous studies on the role of MDH2 in cancer. For instance, Liu et al. [22] reported that silencing MDH2 inhibited the proliferation of prostate cancer cells. Similarly, Pei et al. [34] confirmed that MDH2 silencing inhibited mitochondrial respiration and ovarian cancer cells. Collectively, these studies, including our own, suggest that MDH2 functions as an oncogene in breast cancer and may represent a druggable target for its treatment.

Despite the emerging evidence of MDH2’s role in cancer, the underlying mechanisms by which MDH2 promotes breast cancer growth remain poorly understood. To identify the primary drivers of this oncogenic effect, we performed transcriptome analysis on MDA-MB-231 cells overexpressing MDH2. This approach enabled us to investigate the molecular mechanisms of MDH2 in breast cancer progression on a genome-wide scale with high sensitivity. Our analysis revealed that modulating MDH2 expression significantly impacts the genomic landscape of breast cancer cells. Subsequent pathway analysis using KEGG and GSEA identified the PI3K-Akt and mTOR signaling pathways as potential downstream targets of MDH2. These pathways are well-established regulators of cell proliferation and differentiation, and their activation has been implicated in the development of various human tumors [35,36]. Therefore, we hypothesize that MDH2 may promote breast cancer development by activating these pathways.

In addition to its role in tumor growth, our research also uncovered that MDH2 regulates cell motility, particularly cell migration, which is a critical step in breast cancer progression [11]. To elucidate the role of MDH2 in metastatic breast cancer cells, we performed MDH2 depletion in the highly metastatic MDA-MB-231 breast cancer cell line. Our findings demonstrated that suppressing MDH2 inhibited cell migration and reversed the EMT process. Further transcriptomic analysis revealed that MDH2 overexpression altered signaling pathways known to modulate tumor metastasis, including ribosome biogenesis [37], PI3K-Akt [38], NF-kappa B [39], and Rap1 [40] signaling pathways. Moreover, changes in MDH2 expression led to alterations in various metastasis-associated downstream genes. Specifically, we observed up-regulation of genes that drive breast cancer metastasis (IL13RA2 [41], MMP3 [42], PTGS1 [43], SYK [44], VCAN [45], and FLT1 [46]) and downregulation of metastasis-associated suppressor genes (NOTCH3 [47], and HTRA3 [48]). Collectively, these findings suggest that MDH2 may promote tumor metastasis by modulating these pathways and genes.

Numerous studies have established that the aberrant metabolism, particularly glycolysis, of cancer cells fuels their proliferation and metastasis by providing essential energy sources such as glucose and ATP [49,50]. Our research indicates that MDH2-silenced cells exhibit reduced glucose consumption and ATP production compared to control cells. This observation aligns with previous reports that MDH2 regulates the NAD/NADH ratio to sustain cellular glycolysis [51]. Based on these findings, we hypothesize that inhibiting MDH2 could modulate tumor growth and metastasis by disrupting the NAD/NADH equilibrium and malate-aspartate transport between the cytoplasm and mitochondria, thereby altering the energy metabolism of cancer cells.

Metabolomics offers a powerful tool for precisely investigating disease diagnosis and pathogenesis. Therefore, identifying the metabolites involved in MDH2’s regulation of breast cancer development is crucial. Our study reveals that altered MDH2 expression significantly modifies 62 metabolites in breast cancer, primarily amines, amino acids, nucleosides, and other classes. Although the functions of most metabolites in tumors remain unclear, our findings suggest that MDH2, as a mitochondrial metabolic enzyme, substantially alters the metabolic pattern of cancer cells and influences tumor progression. Notably, MDH2 expression promotes adenosine production and inhibits linoleic acid synthesis in breast cancer. These metabolites are known to modulate the immune response of tumors. Adenosine is a key component of the adenosinergic pathway, which is implicated in cancer immunosuppression; thus, targeting and inhibiting adenosine production could be advantageous for cancer immunotherapy [52]. Linoleic acid, identified as a primary positive regulator of CD8(+) T cell activity, enhances the anti-tumor potency of CD8(+) T cells both *in vitro* and *in vivo* and is currently being explored as an enhancer of T cell therapies in oncology [53].

Accumulating evidence indicates that aberrant metabolism in cancer not only sustains tumorigenesis and survival through cancer signaling but also significantly affects anti-tumor immune responses by releasing metabolites that influence the tumor immune microenvironment [54–56]. Moreover, tumor-infiltrating immune cells, a critical component of the tumor microenvironment, are closely associated with the development, progression, and dissemination of malignant tumors [57,58]. Consequently, investigating the relationship between MDH2 expression and immune infiltration in breast cancer is imperative. Our analysis shows that the levels of multiple immune cell infiltration are substantially negatively correlated with MDH2 expression, and these levels of significantly impact the survival outcomes of breast cancer patients with high or low MDH2 expression. Additionally, patients with high MDH2 expression exhibit reduced immune and stromal scores. Therefore, we propose that MDH2, which is implicated in cancer metabolism, influences breast cancer development by modifying the tumor immune microenvironment through the release of metabolites such as adenosine and linoleic acid.

Given the putative pro-cancer function of MDH2, developing drugs that modulate the activity may offer a novel therapeutic strategy for breast cancer. As previously mentioned, MDH2 catalyzes the interconversion of malate and oxaloacetate using NAD/NADH as a cofactor [15]. Our *in silico* research suggests that designing small-molecule inhibitors based on the NADH-binding tunnel of MDH2 could inhibit its catalytic activity by competing for NADH binding. Moreover, considering MDH2’s role in tumor immunity, combining small-molecule MDH2 inhibitors with immune checkpoint inhibitors may enhance the efficacy of tumor immunotherapy. Several small-molecule MDH2 inhibitors have been successfully developed, expanding the scope of novel cancer metabolism and tumor growth therapies [23,25].

Despite the significant findings of this study, several limitations remain. Although multi-omics analyses suggest that the PI3K–Akt pathway and the adenosine–linoleic acid axis may act as downstream effectors of MDH2, their direct targets and dynamic metabolic flux still require precise validation by techniques such as chromatin immunoprecipitation sequencing (ChIP-seq), isotope tracing, and single-cell technologies. Future work should integrate large-scale clinical cohorts and multidimensional omics platforms to systematically elucidate the clinical translational potential of MDH2.

## 5 Conclusion

In summary, our comprehensive investigation has firmly established MDH2 as a novel oncogene in breast cancer. *In vitro* studies have revealed that silencing MDH2 effectively curtails the proliferative and migratory capacities of breast cancer cells. Conversely, *in vivo* experiments have demonstrated that overexpression of MDH2 robustly drives the growth of breast cancer xenograft tumors. Through meticulous transcriptomic and metabolomic analyses, we have uncovered that alterations in MDH2 expression elicit profound changes in the transcriptomic landscape and metabolic profile of breast cancer cells. These discoveries not only shed new light on the intricate molecular mechanisms underlying breast cancer progression but also offer valuable insights for enhancing the prognosis and therapeutic strategies for breast cancer patients. Importantly, our findings significantly broaden the scope for developing innovative cancer therapies that specifically target cancer metabolism and tumor growth, thereby holding promise for more effective and targeted treatments in the future.

## Supplementary Materials









## Data Availability

All data supporting the findings of this study are available within the paper and its Supplementary Information. Publicly available data were obtained from published TCGA, GTEx, HPA, and TIMER.

## Supplementary Materials

Supplementary file 1: List of 282 differentially expressed genes identified by transcriptomics; Supplementary file 2: List of differential metabolites identified by primary metabolite profiles from mass spectrometry (positive ion mode); Supplementary file 3: List of differential metabolites identified by primary metabolite profiles from mass spectrometry (negative ion mode); Supplementary file 4: List of 62 secondary metabolites identified by metabolomics. The supplementary material is available online at https://www.techscience.com/doi/10.32604/or.2025.068119/s1.

## Abbreviations

MDH2: Malate Dehydrogenase 2
TCGA: The Cancer Genome Atlas
HPA: Human Protein Atlas
TIMER: Tumor Immune Estimation Resource
EMT: Epithelial-Mesenchymal Transition
BRCA: Breast Invasive Carcinoma
TCA: Tricarboxylic Acid
ATP: Adenosine-5^′^-triphosphate
NAD: Nicotinamide Adenine Dinucleotide
NADH: Nicotinamide Adenine Diphosphate Hydride
GTEx: Genotype-Tissue Expression
IHC: Immunohistochemical
OS: Overall Survival
DSS: Disease-Specific Survival
TPM: Transcripts Per Million
ROC: Receiver Operating Characteristic
ssGSEA: Single-Sample Gene Set Enrichment Analysis
DMEM: Dulbecco^′^s Modified Eagle Medium
MEM: Minimal Essential Medium
MepiCM: Mammary Epithelial Cell Medium
RIPA: Radioimmunoprecipitation Assay
BCA: Bicinchoninic Acid
FBS: Fetal Bovine Serum
SDS-PAGE: Sodium Dodecyl Sulfate-Polyacrylamide Gel Electrophoresis
PVDF: Polyvinylidene Fluoride
PI: Propidium Iodide
ECL: Enhanced Chemiluminescence
TBST: Tris-Buffered Saline with Tween 20
NGS: Next-generation Sequencing
GC-MS: Gas Chromatography-Mass Spectrometry
LC-MS: Liquid Chromatography-Mass Spectrometry
VIP: Variable Importance in Projection
SD: Standard Deviation
CHOL: Cholangiocarcinoma
COAD: Colon Adenocarcinoma
BLCA: Bladder Urothelial Carcinoma
ESCA: Esophageal Carcinoma
KICH: Kidney Chromophobe
LIHC: Liver Hepatocellular Carcinoma
LUAD: Lung Adenocarcinoma
LUSC: Lung Squamous Cell Carcinoma
PRAD: Prostate Adenocarcinoma
READ: Rectum Adenocarcinoma
STAD: Stomach Adenocarcinoma
UCEC: Uterine Corpus Endometrial Carcinoma
KIRC: Kidney Renal Clear Cell Carcinoma
PCPG: Pheochromocytoma and Paraganglioma
THCA: Thyroid Carcinoma
ER: Estrogen Receptor
PR: Progesterone Receptor
AUC: Area Under the Curve
DEGs: Differentially Expressed Genes
KEGG: Kyoto Encyclopedia of Genes and Genomes
